# Serous Microcystic Cystadenocarcinoma of the Pancreas with Synchronous Liver Metastases: Clinical Characteristics and Management

**DOI:** 10.7759/cureus.7707

**Published:** 2020-04-17

**Authors:** Dimitrios Massaras, Eirini V Pantiora, John Koutalas, Elias C Primetis, Georgios P Fragulidis

**Affiliations:** 1 Surgery, Aretaieio Hospital, National and Kapodistrian University of Athens School of Medicine, Athens, GRC; 2 Anesthesiology, Aretaieio Hospital, National and Kapodistrian University of Athens School of Medicine, Athens, GRC; 3 Radiology, Tiefenau Hospital, Bern, CHE; 4 2nd Department of Surgery, “Aretaeio” Hospital, National and Kapodistrian University of Athens School of Medicine, Athens, GRC

**Keywords:** cystadenocarcinoma, pancreas, sunitinib, microcystic, serous cystic neoplasm, liver metastasis, indications for surgery, digestive surgery

## Abstract

Serous cystadenocarcinoma of the pancreas is a rare but well-established entity. The origin and evolution of this disorder remain unclear, but even metastatic cases have an excellent prognosis. These tumors are very similar to benign serous cystic neoplasms (SCNs) of the pancreas, except that they tend to be larger, are locally invasive, and present distant metastasis. The most frequent local invasion is adjacent vessels, spleen, stomach, and duodenum. The most common site of distant metastasis is the liver. Diagnosis via imaging as well as pathology examination may be misguided due to atypical characteristics of the tumor. In fact, in some, the diagnosis of malignancy was established only after metastases were detected. We present a 60-year-old female patient with malignant serous microcystic cystadenocarcinoma of the pancreas and liver metastasis that was initially misdiagnosed as a metastatic renal cell carcinoma. The patient underwent tumor resection and liver metastasectomy and she is currently doing well after three years of follow-up, with no tumor recurrence or new metastatic liver nodules based on imaging findings.

## Introduction

Serous cystic neoplasms (SCNs) of the pancreas are rare cystic tumors that account for less than 1% of all primary pancreatic lesions. They are often diagnosed at the age of 50-70 years, and the incidence is higher in women (70%) than in men [1]. Although they are mainly considered as a benign disease, they differ markedly in terms of their malignant potential, spanning a spectrum of clinical behavior that includes entirely benign, potentially malignant, and frankly malignant characteristics [2]. There are five recognized subtypes of SCNs of the pancreas identified as cystadenomas: serous microcystic cystadenomas, serous oligocystic cystadenomas, serous solid cystadenomas, von Hippel-Lindau (vHL) disease associated cystadenomas, and serous cystadenocarcinoma as their malignant counterpart [3].

Serous cystadenocarcinomas are extremely rare, with estimates of malignant conversion of cystadenomas ranging between 1% and 3%. The histological characteristics of serous cystadenocarcinoma are indistinguishable from its benign counterparts, making the presence of invasion or metastasis the exclusive distinguishing characteristic between them [4]. As a result, the World Health Organization established the definition of serous cystadenocarcinoma in 2010, and outlined malignancy as the presence of distant metastases regardless of benign-looking histology features [5]. In addition to distant metastases, many studies have likewise classified serous cystadenomas that invade surrounding organs as serous cystadenocarcinoma. To end this up, currently direct invasion of the adjacent organs and the presence of distant metastases are hallmarks of malignant SCNs of the pancreas.

Diagnosis is primarily based on imaging findings; however, there are many atypical radiologic characteristics of SCNs that make the correct diagnosis challenging. Microscopically, furthermost serous cystadenocarcinomas are cyst-forming neoplasms composed of cuboidal epithelial cells rich in glycogen with no elements of cellular atypia separated by thin vessel-containing fibrous septae, virtually indistinguishable from those of serous cystadenomas. Besides, examples of metastatic ovarian clear cell adenocarcinoma and clear renal cell carcinoma were mistaken for serous microcystic cystadenocarcinomas [6]. In this report we present a case of pancreatic serous microcystic cystadenocarcinoma with invasion of the spleen, left adrenal gland and metastasis into the liver, originally misdiagnosed and treated as metastatic renal carcinoma with the tyrosine kinase inhibitor sunitinib.

## Case presentation

**Figure 1:**
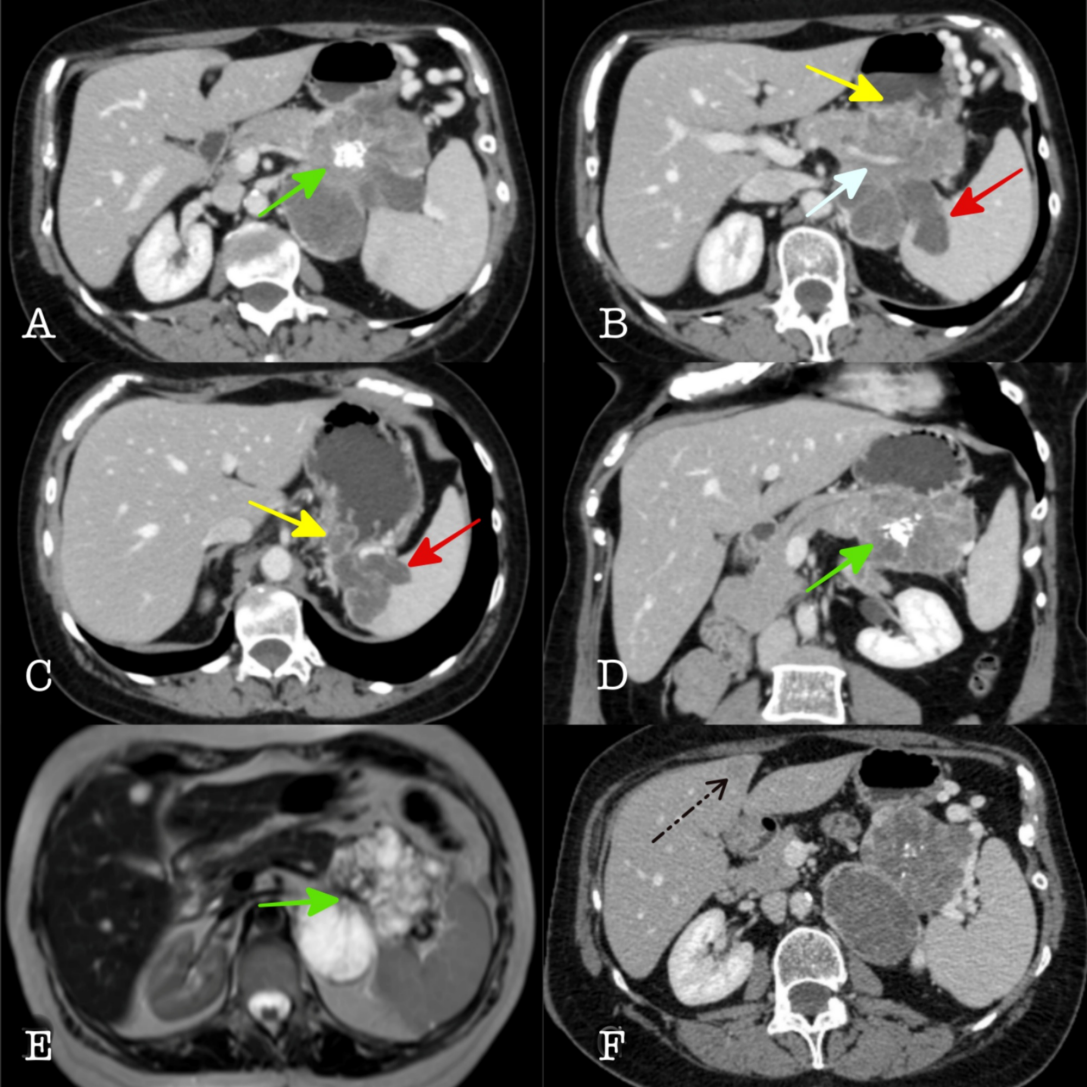
(A,B,C,F) Contrast enhanced axial CT slices, D) Oblique coronal CT reconstruction, and E) T2 axial MRI show a large lobulated, multicystic mass with central calcified scar. Multicystic appearance and central calcified scar (green arrows) resemble the imaging features of the more common benign serous cystadenoma (microcystic adenoma), however, invasion of splenic vessels (white arrow), spleen (red arrows), stomach wall (yellow arrows), and liver metastases (black arrow) indicate a more aggressive, malignant lesion.

A 60-year-old female patient with a history of radical hysterectomy for endometrioid carcinoma (FIGO II) presented with a tumor in the tail of the pancreas during her follow-up and she was referred to our hospital for additional work up. MRI scan showed a pancreatic tumor that measured 8.6 cm x 7 cm x 9 cm infiltrating the surrounding tissues (spleen, left kidney, and left adrenal gland) and a metastatic lesion (segment IV) in the liver as can be seen in Figure 1.

**Figure 2:**
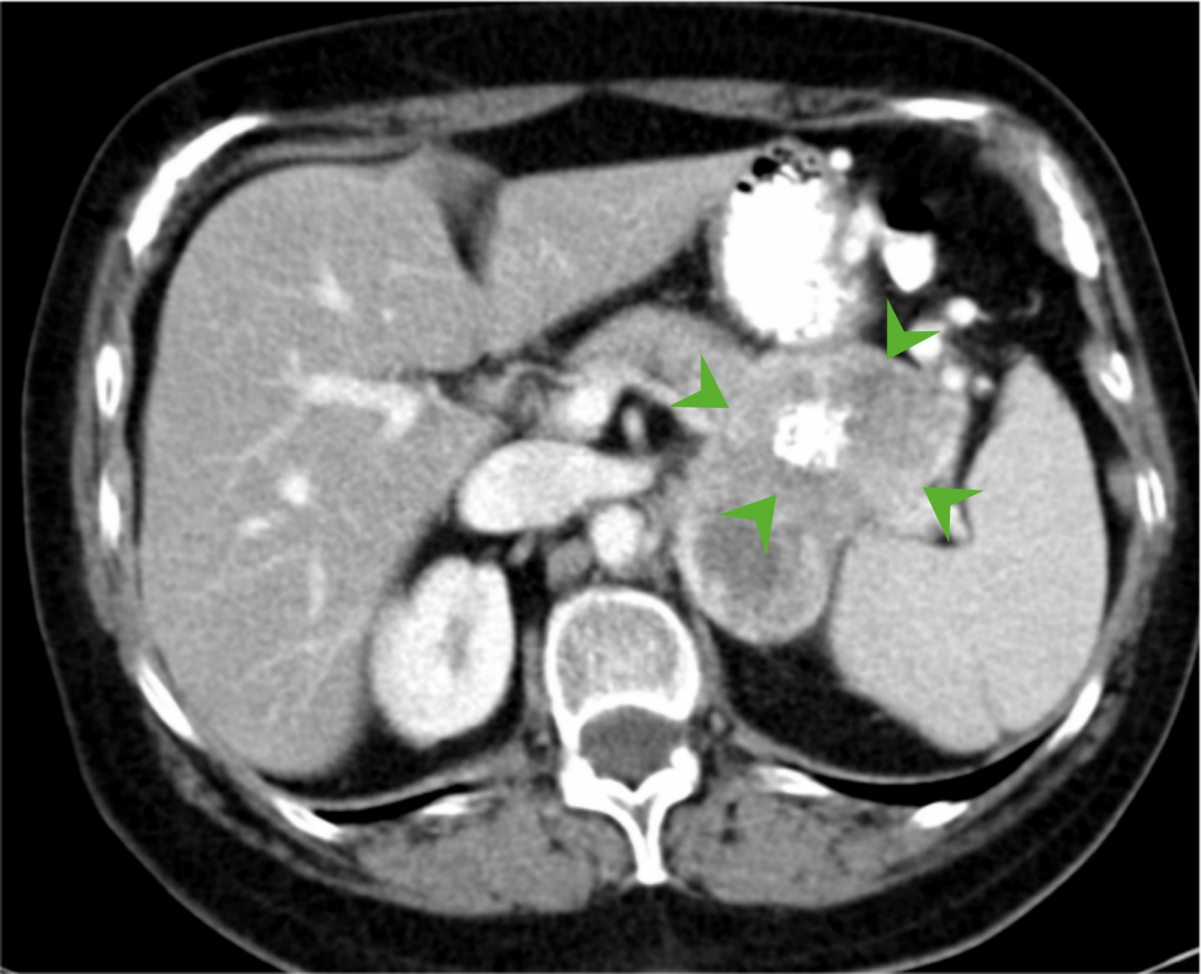
Axial contrast enhanced CT scan 11 months later. The mass has decreased in size (arrowheads).

Tumor markers carcinoembryonic antigen, carbohydrate antigen 19-9, and serum chromogranin-A were within the normal range. An endoscopic ultrasound (EUS) and fine needle aspiration (FNA) of the tumor were performed and the cytology described the lesion as clear cell carcinoma of the kidney. The patient was referred to the medical oncology department with a provisional diagnosis of renal cell tumor and a multi-targeted receptor tyrosine kinase inhibitor (sunitinib) 50 mg/day was initiated. After four cycles of sunitinib, a follow-up CT scan demonstrated a decrease in the size of the tumor (6.3 cm x 4.3 cm x 3 cm) (Figure 2).

**Figure 3:**
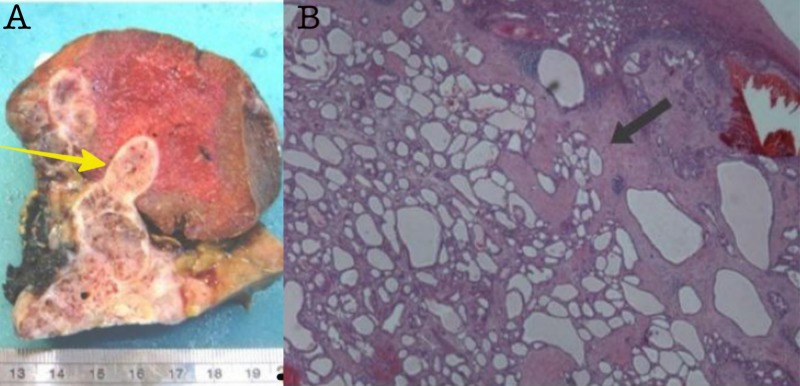
Images of the interface of serous neoplasm with splenic parenchyma. (A) Gross pathology photograph of the tumor with fine external lobulations and mutiple thin enhancing internal septations. (B) Microscopic view of the surgical specimen shows the spleen infiltrated by the tumor (arrow). The tumor is composed of multiple cysts lined by cuboidal cells with clear cytoplasm (H&E x20).

**Figure 4:**
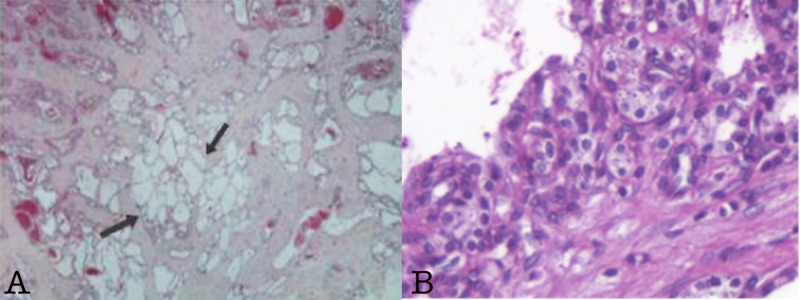
Pathology features of metastatic microcystic serous cystadenocarcinoma to the liver. (A) Metastatic microcystic serous cystadenocarcinoma to the liver (H&E x100) with the presence of clear cells. The pathology features were similar to those of the primary tumor. (B) Periodic acid-Schiff stain highlighting intracytoplasmic glycogen granules.

Next, following the decision of multidisciplinary meeting, the patient underwent exploratory laparotomy. A distal pancreatectomy en bloc with the spleen and left kidney was performed in addition to liver metastasectomy. No signs of other metastases were recognized during surgery. The histology report described the tumor as serous microcystic neoplasm of the pancreas and it was classified as serous cystadenocarcinoma due to the infiltration of the spleen, left adrenal gland, and liver metastasis (Figures 3 and 4A).

All resection margins and 11 lymph nodes were uninvolved. The kidney was in close proximity with the tumor without being infiltrated. The neoplastic cells were with clear cytoplasm and glycogen rich by periodic acid-Schiff stain (Figure 4B). No adjuvant chemotherapy was administered and at last follow-up three years post-operatively, the patient was asymptomatic without clinical or radiographic evidence of recurrent disease.

## Discussion

Serous cystic neoplasms of the pancreas had been classified by Compagno and Oertel in 1978 as benign epithelial neoplasms with no malignant potential. They were characterized by a repetitive cuboidal epithelial cell proliferation with marked cytoplasmic clearing due to intracellular glycogen [7].

The majority has microcystic variety, which is synonymous with ‘‘serous cystadenoma’’ of the pancreas. This term has been used historically to refer to the broader class, which consists of several more uncommon histologic variants included in the context of SCNs. Although these tumors had been considered initially as benign, reports have documented SCNs of the pancreas with malignant behavior such as invasion of surrounding organs or metastasis in the path of “serous cystadenocarcinoma” [8-9]. The growing evidence of malignant transformation SCNs of the pancreas into cystadenocarcinoma is suspected by increase in size, concept of adjacent organ invasion, distant metastases, and histological features suggestive of vascular/lymphatic or neural invasion. Thus, the worrying features include rapid enlargement, onset of new symptoms clinically, and new radiological findings related to the tumor. Microscopically, cystadenocarcinomas are identical to serous cystadenomas with the only distinguishing feature being gross or microscopic evidence of invasiveness. Indeed, in some as in our case, the diagnosis of malignancy is established only after metastases to the liver are detected [1].

The SCNs occur more commonly in females and manifest with no specific symptoms. Up to one-third of the patients are diagnosed incidentally during investigation for nonrelated reasons, as the present case, which was diagnosed during her follow-up for. They are often localized in the body or tail of the pancreas (50%-75%) and most often manifest with abdominal, flank, or epigastric pain. Although rare cases present with jaundice or pancreatitis, these are unusual, even for SCNs occurring in the head of the pancreas [2].

Diagnosis is primarily based on imaging findings, however, there are many atypical radiologic characteristics of SCNs that make the correct diagnosis challenging. The prevalence of pancreatic cystic lesions on abdominal imaging has been reported to be between 2.6% and 19.6% and overall an accurate radiologic diagnosis was rendered in only 14% of cases [10]. Though MRI is frequently used for characterization of cystic pancreatic lesions, CT remains the first-line imaging modality due to more widespread availability. Serous cystadenomas do not usually communicate with the pancreatic duct. Fine external lobulations are a common and characteristic feature and the presence of fibrous central scar, with or without a stellate pattern of calcifications seen in 30% of cases, is highly specific for serous cystadenoma [11]. Indeed, the imaging findings of the malignant SCNs of the pancreas are basically identical to those of the benign counterparts, except for malignant behavior such as local invasion or presence of distant metastases [12]. Thus, the preoperative differentiation between benign and malignant SCNs remains difficult. The risk of malignancy was significantly increased in patients with a cyst >3 cm and with the presence of a solid component. Therefore, patients with a pancreatic cyst with concerning features on CT or MRI should undergo further evaluation with EUS and FNA to confirm the diagnosis [13]. However, definitive diagnosis on FNA biopsy is possible in 25% of cases due to the low sample cellularity and contamination resulting from an endoscopic approach. The combination of cytology studies with concurrent cyst fluid sampling may help to enhance diagnostic specificity and sensitivity. The cyst fluid in serous cystadenoma generally shows low carcinoembryonic antigen and amylase [2].

Most serous cystadenocarcinomas are solid, well circumscribed, and lobulated masses which range in size from 0.1 cm up to more than 25 cm. In the microcystic variant innumerable small cysts give the appearance of a sponge or honeycomb, with centrally located area of fibrotic scar in a subset of cases. In the solid variant the lack of cystic spaces on gross inspection often gives the initial impression of a neuroendocrine or solid pseudopapillary neoplasm [2]. Microscopically, the cells are uniform, round to polygonal, and have clear or pale eosinophilic cytoplasm with well-defined borders. Special stains highlight the abundant intracytoplasmic glycogen and absence of stainable mucin [3, 14]. It is noteworthy to mention that in a recent review, Reid et al. reported an association of SCNs in resection specimens with other neoplasms of the uninvolved pancreas in 13%. The presence of neuroendocrine tumors was the most common concurrent neoplasm in 44% of these cases [10].

The differential diagnosis includes other cystic lesions of the pancreas, such as intraductal papillary mucinous and mucinous cystic neoplasm, along with typically solid neoplasms, such as neuroendocrine tumor, solid pseudopapillary neoplasm, and pancreatic ductal adenocarcinoma. Pancreatic ductal adenocarcinoma may mimic the solid variant or exhibit a deceptively cystic-appearing architecture. In general, the low-grade cytology along with the absence of malignant characteristics, such as perineural or lymphovascular invasion, is perhaps most critical to distinguishing SCNs from malignant tumors. Although immunohistochemistry is often not required recognition of gene expression patterns in SCNs can help differential diagnosis, regrettably a lesson learned from the present case. In keeping with their epithelial nature, the neoplastic cells of SCNs of the pancreas invariably express epithelial membrane antigen and pan-cytokeratins. Among cytokeratins, cytokeratin 7 (CK7), CK18, and CK19 are expressed, whereas CK14, CK17, and CK20 are not. Unrivalled, the expression of both GLUT1 and carbonic anhydrase IX in a predominantly membranous pattern, along with the association of SCNs with vHL mutations, have drawn comparisons to clear cell renal cell carcinoma and capillary hemangioblastoma. This immunohistochemical overlap warrants diagnostic confirmation with additional markers when considering a metastatic renal carcinoma, particularly in the patient with a history of vHL [2]. In addition, an assay of vascular endothelial growth factor (VEGF), a molecule involved in the clear cell tumor genesis pathway, of which serous neoplasms are a prototype, was recently found to be highly sensitive and specific for this entity [10].

There have been a limited number of reports of chemotherapy or radiotherapy for serous cystadenocarcinomas with uncertain results though could be considered in unresectable cases [14]. Yet, the unexpected result of the misjudged treatment that in long period received our patient raises the question of a possible therapeutic effect of the anti-VEGF multi-targeted receptor tyrosine kinase inhibitor (sunitinib) in this type of cancer.

Given the lack of clear and convincing data, management of patients with SCNs should be individualized, unless they exhibit aggressive pattern or unspecific features in terms of cystadenocarcinoma. Most cases of SCNs had a slowly progressive clinical course as the malignant potential is very low. Distinction of this benign entity from other solid clear cell neoplasms of the pancreas is important because the differential includes malignant and aggressive neoplasms. Follow-up imaging every two years is recommended for serous cystadenomas between 2 and 3 cm. The growth patterns for serous cystadenomas were similar regardless of initial size with estimated doubling time of 12 years. Doubling of size in less than 12 years should be considered for resection regardless of initial reported size [15]. Nevertheless, surgical management should be commenced to a selected group of patients in whom the follow-up radiology imaging shows considerable growth and worsening of symptoms [12-13, 16]. In these cases, resection may be indicated, despite the lack of objective evidence for malignancy obtained from preoperative imaging, endoscopy, and biopsy [4].

Despite the aggressive locoregional disease, serous cystadenocarcinomas generally have an unexpected remarkable prognosis. A recent study and reappraisal of the published cases reporting ‘‘malignant’’ SCNs of the pancreas, found that none of these cases showed true malignant behavior in the form of metastatic disease [10]. Even cases in which liver involvement is identified, they do not show any evidence of malignancy by histology and true malignant behavior is exceedingly uncommon [2]. Reports in the literature describing serous neoplasms metastatic to the liver were limited, and of these 10 (59%) had metachronous and 7 (41%) had synchronous liver serous neoplasms with one case being clinically suspicious for vHL disease [10]. For cases with liver involvement, which naturally had been classified as liver “metastasis”, Reid et al. propose an alternate possibility of “multifocal” disease rather than true metastasis at least for some of these cases, because of the following reasons: (1) none of them were reported to have further malignant behavior or fatality; (2) there was neither metastasis to organs other than the liver, nor was there remote nodal metastasis, as most epithelial tumors that metastasize to the liver also typically metastasize to remote lymph nodes first; (3) the occurrence of a liver SCN without a pancreatic “primary” proves that hepatic SCNs can occur independently; (4) there is the phenomenon of synchronous and metachronous hepatic and pancreatic affliction by other cystic lesions including vHL-associated polycystic disease and mucinous cystic neoplasm; and (5) all reported cases had a bland cytology and lacked histomorphologic evidence of malignancy. Therefore, it is conceivable that the simultaneous occurrence of SCNs in the liver may, at least in some cases, represent multifocality (synchronous disease) rather than true metastasis [10].

## Conclusions

Serous cystic neoplasms of the pancreas are rare tumors, usually incidentally diagnosed that may present invasion of surrounding organs or distant metastasis, in the term of cystadenocarcinoma. Follow-up imaging every two years is recommended for serous cystadenomas between 2 and 3 cm. Cystadenocarcinoma is a slowly growing neoplasm with a relatively good prognosis even in the presence of metastases. Therefore, aggressive surgical management is still considered as the main treatment option for this rare disease. Furthermore, although there is a significant progress in our understanding of the natural history of SCNs, their potential for clinically malignant behavior is still under debate.
